# P08 Antibiotic prescribing practices among Belgian dentists: 2014–23

**DOI:** 10.1093/jacamr/dlaf230.015

**Published:** 2025-12-04

**Authors:** Moira Kelly, Roos Leroy, Thomas Struyf, Boudewijn Catry, Lucy Catteau

**Affiliations:** Department of Epidemiology and Public health, Sciensano, Brussels, Belgium; Belgian Health Care Knowledge Centre (KCE), Brussels, Belgium; Academic Centre for Family Medicine, KU Leuven, Leuven, Belgium; Department of Epidemiology and Public health, Sciensano, Brussels, Belgium; Faculty of Medicine, Université Libre de Bruxelles, Brussels, Belgium; Department of Epidemiology and Public health, Sciensano, Brussels, Belgium; Faculty of Medicine and Pharmacy, Université de Mons (UMons), Mons, Belgium

## Abstract

**Background:**

In 2016, dentists accounted for 5.8% of all outpatient antibiotic prescriptions in Belgium. This study evaluated antibiotic prescribing trends in dental practice from 2014 to 2023 in Belgium and explored the influence of prescriber demographics (age, sex and specialty) and of the COVID-19 pandemic on prescribing behaviour.

**Methods:**

Data on reimbursed systemic antibiotics (ATC J01) dispensed by community pharmacies were obtained from the Belgian National Institute for Health and Disability Insurance (NIHDI) and analysed to evaluate prescriptions issued by dentists from 2014 to 2023. Antibiotic use was quantified in DDDs and expressed as DDD per 1000 insured beneficiaries per day (DBD). Data on dentist demographics (age, sex, specialty) and professional activity (annual number of consultations for 2018–22) were also obtained from NIHDI and used to assess prescribing behaviour.

**Results:**

Total antibiotic prescriptions by Belgian dentists (measured in DDD) increased by 13.5% from 2014 to 2023. Despite a 3.1% reduction in DDD in 2020, the volume of J01 antibiotics prescribed per consultation increased during that year. The proportion of total outpatient J01 antibiotic deliveries prescribed by dentists increased from 4.9% in 2014 to 6.3% in 2023, with a peak of 7.3% in 2021. Male and older dentists consistently prescribed more antibiotics than their younger and female counterparts. Following the introduction of national antibiotic guidelines for dentistry in 2020 we observed limited change in the proportion of recommended antibiotics prescribed.

**Conclusions:**

Antibiotic prescribing among Belgian dentists is influenced by various factors, including prescriber characteristics and external disruptions such as the COVID-19 pandemic. Opportunities remain to optimize prescribing practices, and efforts should focus on encouraging guideline-based antibiotic use only when necessary.

